# Overcome the fragmentation in online propaganda literature: the role of cultural and cognitive sociology

**DOI:** 10.3389/fsoc.2023.1170447

**Published:** 2023-07-11

**Authors:** Valentina Nerino

**Affiliations:** ^1^Interdisciplinary Centre for Gender Studies (ICFG), University of Bern, Bern, Switzerland; ^2^Department of Sociology, University of Trento, Trento, Italy

**Keywords:** online propaganda, social media, persuasion, information processing, culture, cognition

## Abstract

Evidence concerning the proliferation of propaganda on social media has renewed scientific interest in persuasive communication practices, resulting in a thriving yet quite disconnected scholarship. This fragmentation poses a significant challenge, as the absence of a structured and comprehensive organization of this extensive literature hampers the interpretation of findings, thus jeopardizing the understanding of online propaganda functioning. To address this fragmentation, I propose a systematization approach that involves utilizing Druckman's Generalizing Persuasion Framework as a unified interpretative tool to organize this scholarly work. By means of this approach, it is possible to systematically identify the various strands within the field, detect their respective shortcomings, and formulate new strategies to bridge these research strands and advance our knowledge of how online propaganda operates. I conclude by arguing that these strategies should involve the sociocultural perspectives offered by cognitive and cultural sociology, as these provide important insights and research tools to disentangle and evaluate the role played by supra-individual factors in the production, distribution, consumption, and evaluation of online propaganda.

## 1. Introduction

The emergence of the Internet has revolutionized the way political information is created, disseminated, and consumed, particularly through Social Networking Platforms (SNPs), which have become a crucial arena for political communication. Given their centrality, the circulation on such platforms of political content that appears dubious in regard to its factuality, approval rates, and political motive has raised widespread concern. Numerous investigations have, indeed, uncovered the widespread presence of political content on SNPs that masquerades as reliable, neutral information—though it aims to discredit opposing viewpoints rather than serving an informative purpose (Tucker et al., 2018)—and it is frequently amplified through the systematic use of automated tools and impersonation of accounts (Woolley and Howard, 2018).

Such findings have urged scholars to extensively address this alarming phenomenon—which in this paper is referred to as online propaganda—leading to a thriving yet quite disconnected body of literature. This fragmentation can be attributed to the interdisciplinary nature of the research itself as well as to the complexity and multidimensionality of the phenomenon it addresses. In fact, scholars investigating online propaganda not only employ various theoretical and methodological approaches, but they also focus on different dimensions of the phenomenon, contributing to the disconnected nature of the literature. This poses a significant challenge, as the absence of a structured and comprehensive organization of the extensive literature generated thus far hampers the interpretation and systematization of findings in relation to previous research within this field. As a result, the overall comprehension of the phenomenon is jeopardized.

To address this fragmentation, I propose a novel approach to analyze and structure the existing body of literature on online propaganda. This approach involves utilizing Druckman's (2022) Generalizing Persuasion (GP) Framework as a unified interpretative framework to organize this scholarly work. By adopting this approach, I argue that it is possible to systematically identify the various strands within this field of study and recognize their respective shortcomings. Additionally, employing such strategy enables the formulation of new strategies to bridge these research strands and advance our comprehension of how online propaganda operates.

Hence, this paper is organized as follows: firstly, it presents a theoretical definition of the phenomenon under investigation and introduces the GP Framework as a mean to consolidate the fragmented literature; secondly, it examines the different strands within the literature using this framework; finally, it addresses the limitations of each strand and suggests approaches to overcome them while also establishing connections between the various strands. In particular, arguments are made in favor of incorporating sociocultural perspectives in both the theoretical conceptualization and empirical evaluation of online propaganda functioning as an effective method to bridge the literature.

## 2. Defining online propaganda

Propaganda is still a much-debated term in the literature, which is often applied in very diverse contexts with different meanings and implications. The conceptual and, thus, terminological entropy characterizing the literature on propaganda is related to the multidimensional *nature of the phenomenon*—Do we intend propaganda as a communication practice, a public opinion issue, or a “more general” political phenomenon?—as well as its complex *relation with persuasion*—Is one a subcategory of the other or are there structural differences between the two?

Drawing from Jowett and O'donnell (2018), in this paper propaganda is intended as a specific *class of communication* that involves two actors—a sender and a receiver—who, through a process of symbolic interaction, use information in an attempt to share meaning. Although propaganda has important similarities with persuasion—highlighted by the presence of persuasive communication elements—it differs from the latter in one crucial aspect. While persuasive and propagandist communication share the same aim—i.e., influence a targeted audience into voluntarily adopting a point of view and/or a behavior favoring the sender's interest—the former is overt about its persuasive intentions, while the latter is not (Jowett and O'donnell, 2018). Indeed, propaganda wants to pass as informative communication—whose only purpose is to create mutual understanding of data, concepts, and ideas that are considered to be accurate and fact-based. Therefore, the major difference between persuaders and propagandists lies in the fact that the former do not want to appear as informers, while the latter do (Jowett and O'donnell, 2018). Building on these considerations, propaganda can be therefore defined as a type of communication with a concealed persuasive aim, which is pursued in a systematic and organized way—i.e., with a clear and deliberate political strategy (Jowett and O'donnell, 2018).

To depict *online* propaganda, however, a further specification is required. Indeed, the Internet—and, in particular, SNPs—have profoundly altered “classical” top-down communication models, where sender-receiver roles were usually static and unidirectional (Wanless and Berk, 2021). In fact, these platforms have transformed their users into productive consumers—or *prosumers* (Fuchs, 2014)—who, rather than being passively exposed to information, have an active role in its production and circulation. This aspect is of crucial importance for online propaganda, as it makes the traditional distinction between “propagandist” and “targeted audience” blurred, transforming social media users into potentially active campaigners (Wanless and Berk, 2021). This is why, compared to its traditional form, online propaganda is *participatory*, as it tries to co-opt its targeted audience to actively engage in the spread of its messages (Wanless and Berk, 2021).

This conception of online propaganda is far from being definitive. Nonetheless, it is particularly enlightening as it stresses a fundamental—and yet often overlooked—aspect of this phenomenon, namely the fact that, as a communication practice, online propaganda necessarily involves the interaction between two actors: a sender and a recipient.

Despite this relational aspect, studies addressing online propaganda often tend to focus on either one or the other actor, adopting different theoretical and empirical approaches that are rarely in dialogue with each other. This has produced a thriving yet quite disconnected scholarship, which makes its systematization particularly arduous. The lack of dialogue between such approaches—and their findings—hinders a coherent advancement of the literature, potentially jeopardizing a comprehensive understanding of the phenomenon. To overcome this issue, the following section proposes a systematization of the current scholarship under a common framework—the Generalizing Persuasion (GP) Framework by Druckman (2022). The intent is to evaluate different strands of literature on the same conceptual basis to clearly determine the contribution of each approach as well as its limitations, and later identify potential strategies to overcome existing shortcomings and bridge the two approaches.

## 3. Systematizing a disconnected literature under the GP Framework

The GP Framework (Druckman, 2022) was developed to offer a conceptual tool to systematize and draw generalization from the vast, but highly fragmented, scholarship on persuasion. It has been designed to highlight the sources of variations considered pivotal for the understanding of the phenomenon, in order to easily identify the connection between different studies addressing persuasion. Given the incorporation of elements of persuasive communication into propagandistic communication and the—though covert—persuasive aim of the latter (Jowett and O'donnell, 2018), the deployment of the GP Framework is considered particularly suitable for the systematization task that has been set.

This framework identifies four core elements (or dimensions) in the study of persuasive communication: actors, treatments, outcomes, and settings. Each of them encompasses different components^1^ that serve to better specify the aspects addressed by each dimension. Druckman makes clear that by no means does the GP Framework requires researchers to account for all the dimensions (and all their respective components) identified when investigating persuasive communication, but it rather urges them to be explicit about which elements they study and how, so that dialogue within the literature can emerge and potential contradictions can be overcome.

When assessing the literature on online propaganda by means of the GP Framework, it appears evident that the discontinuity previously mentioned is not only due to different theoretical and methodological traditions, but it is also related to different research goals, which are reflected in the dimensions addressed—and neglected—by the investigations. When accounting for these, two major strands of the literature emerge.

The first—which we could call “supply-side”—is mostly preoccupied with the question of how people get exposed to propaganda content on SNPs, thus focusing on the process and the motivations behind the production, supply, and availability of propaganda—including patterns of exposure to and engagement with such political material (Guess and Lyons, 2020). As such, investigations belonging to this strand tend to study a specific component of the “actors” dimension, namely the “speaker(s)”, completely neglecting the “receiver(s)” component and, consequently, the “outcome” dimension all together^2^.

Conversely, the second—which can be labeled “demand-side”—engages with the study of the effects that exposure to online propaganda produces on its targeted audience, thus concentrating on the mechanisms underlying the persuasion process (Adam-Troian, 2022). Studies interested in this tend to focus on the “receiver(s)” component of the “actors” dimension, overlooking its counterpart (i.e., “speaker(s)”) but thoroughly addressing the “outcome” dimension instead.

In the following sections, these two approaches will be further examined by means of the GP Framework, with the aim of better positioning their contributions within the literature, addressing their limitations, and discussing how these—if not accounted for—may represent important shortcomings for the sustainable development of the literature.

## 4. The supply-side approach: propagandist, their aims and their goals

Scholars adopting a supply-side approach to the study of online propaganda are usually preoccupied with the identification and classification of the political actors involved in this communication practice, the aims underlying their actions, and the strategies implemented to accomplish them—all while considering the specific features of the different political contexts in which such a phenomenon arise^3^. As such, these investigations mostly focus on three dimensions of the GP Framework, namely “actors”, “treatments”, and “settings”, though they do not address all the components these dimensions encompass.

Most notably, they devote most of the attention to the “speaker(s)” components of the “actors” dimension, concentrating research efforts on the identification of the “type(s)” of speakers involved in the production, distribution, and proliferation of propaganda on SNPs, as well as the “motivations” behind their actions. Indeed, this stand of the literature—whose research agenda has been largely stimulated by the Computational Propaganda Project at the Oxford Internet Institute (2023) and the Observatory on Social Media at the Indiana University (OSoMe, 2023)—is mostly preoccupied with uncovering the identity, the location, and the motives of online propagandists, as well as their organizational practices and methods of dissemination (Guess and Lyons, 2020). This work—often complemented by reports from non-academic sources, such as journalists, intelligence agencies, and SNPs themselves—has provided important insights on the characteristics of both online propagandists and the proliferation patterns of their messages.

Studies on the production and supply of online propaganda have uncovered how this is a widespread communication practice implemented by numerous actors around the Globe, who are very diverse in terms of identity, organizational structure, and motives (Woolley and Howard, 2017, 2018; Bradshaw and Howard, 2018). Ranging from state and intelligence agencies (Bastos and Farkas, 2019; Dawson and Innes, 2019) to teenage groups (Kirby, 2016) and political extremists (Marwick and Lewis, 2017), these “speakers” have very different aims for propagating their messages. Some are driven by economic reasons^4^, while others have political aims encompassing social control—in the case of authoritarian states—issue-salience alterations and framing for electoral goals—in democratic regimes (Woolley and Howard, 2017). In terms of organizational structure, these actors also vary greatly, with investigations showing significant differences in terms of capacity, coordination, and resources (Bradshaw and Howard, 2018).

The supply-side literature on online propaganda has also provided important insights into the “treatments” and “settings” dimensions of the phenomenon, by exploring production and dissemination strategies employed by propagandists on SNPs. By mapping propaganda networks (e.g., Ferrara et al., 2016; Benkler et al., 2018; Vosoughi et al., 2018; Ahmed et al., 2020) as well as the type of messages circulating through them (e.g., Howard and Kollanyi, 2016; Rosińska, 2021), researchers have developed a detailed depiction of the online ecosystems where this kind of material proliferates, identifying diffusion patterns as well as techniques adopted for maximizing message propagation. On this latter, they have uncovered the widespread use of automation (often combined with human curation) to enhance the circulation of specific political stances, as documented in numerous studies on *political bots*^5^ (e.g., McKelvey and Dubois, 2017; Woolley and Howard, 2018; Ferrara, 2020). It is interesting to notice that automation, in addition to enhancing dissemination, also serves the purpose to maintain propagandists anonymous throughout the communication process—a crucial aspect in ensuring that also their intentions remain concealed.

Despite a preponderant focus on “speakers”, “settings”, and “treatments”, the supply-side strand also includes contributions that have tried to address the “outcome” dimension of the phenomenon (e.g., Forelle et al., 2015; Woolley and Howard, 2016; Bradshaw and Howard, 2018). However, these studies remain speculative in nature, as authors never empirically assess their postulations on online propaganda effects—which they identify as the “manipulation of public opinion” (Woolley and Howard, 2018). In fact, they do develop some theoretical considerations regarding the macro-processes through which the manipulative power of online propaganda unfolds^6^, but such considerations account only for the alteration of the political narratives on SNPs, failing to explain how the alteration translates into the manipulation of public opinion (Camargo and Simon, 2022). Authors do recognize this issue, attributing its causes to the difficulty of empirically establishing causal claims on effects that transcend the online realm^7^ (Woolley and Howard, 2018). However, I would argue that the problem is first and foremost theoretical and that the empirical challenges identified by these authors are a consequence of it.

As a type of communication, online propaganda involves the *interaction* between two actors: “speakers” and “receivers”. It is in the unfolding of this interaction—and not in the actions of one or the other actor alone—that the mechanisms determining the persuasive result should be sought. It follows that, if “receivers” and their response to online propaganda are systematically neglected^8^ in the postulation of such mechanisms, the assessment of the persuasive outcome becomes virtually impossible.

Therefore, to go beyond postulations that seem to simply assume an automatic link (and not a testable mechanisms) between exposition to propaganda material and a voluntary change of attitudes and/or behavior, direct engagement with propaganda recipients must be envisaged when discussing online propaganda “outcomes”.

## 5. The demand-side approach: exploring online propaganda effects on its targets

Scholars adopting a “demand-side” approach to the study of online propaganda are interested in exploring the effects this communication practice generates in its targeted audience. As such, their investigations tend to focus on the persuasion process(es) triggered by these political messages, with the aim to identify the micro-mechanisms underpinning evaluations and behavior of those exposed to them. Given these research objectives, contributions belonging to this strand of the literature touch on all four dimensions of the GP Framework (i.e., “actors”, “treatments”, “outcomes”, and “settings”), albeit focusing on only some of the components that fall under these dimensions. Indeed, the demand-side scholarship is interested in assessing online propaganda “outcomes” by identifying factors that influence them—namely, “settings”, “treatments”, and characteristics of “receiver(s)”—and explaining their underlying mechanisms—i.e., the “process” through which information is assessed and decisions are formed.

Conversely from their supply-side colleagues, demand-side scholars are not interested in investigating how social media users end up getting exposed to online propaganda, but they are rather concerned with the cognitive mechanisms that are activated once exposure has occurred. It follows that their primary focus is “receivers” and the way they process and respond to online propaganda. Therefore, the “outcomes” they are interested in are those related specifically to these actors, that is, the observable voluntary changes in the “attitudes” and “behaviors” of the recipients.

As previously discussed, it is extremely complex to causally link exposure to online propaganda with offline behavior (e.g., voting, demonstrating, rioting), as “the effects [of online political persuasive content] on electoral outcomes or other behavior have yet to be reliably detected” (Guess and Lyons, 2020, p. 22). Therefore, the outcomes studied usually concern receivers' evaluation of online propaganda material and the (online) response such material elicit. On the one hand, this means assessing users' perceptions of information *credibility*^9^ (e.g., Castillo et al., 2011; Metzger and Flanagin, 2013; Braddock and Morrison, 2020; Wittenberg and Berinsky, 2020), *reliability*^10^ (e.g., Diviani et al., 2015), and *accuracy*^11^ (e.g., Lucassen and Schraagen, 2011; Pennycook et al., 2018). On the other hand, it means investigating online engagement—which is usually operationalized as sharing behavior—prompted by propaganda content (Islam et al., 2020; Pennycook and Rand, 2021; Liang et al., 2022; Song et al., 2023).

To understand whether and how these evaluations and behaviors are altered by online propaganda, researchers have identified and investigated the influence exerted by three main factors: the design of online propaganda messages (i.e., the “treatments”), the features of the information environments in which these messages are circulated and processed (i.e., the “settings”), and the characteristics of those exposed to such messages (i.e., the “receivers”).

Studies addressing the first factor investigate how different message features that are commonly employed to evaluate information validity and salience—such as source (e.g., Ecker et al., 2022), endorsement (e.g., Metzger et al., 2010; Metzger and Flanagin, 2013), emotional salience (e.g., Ali and Zain-ul-abdin, 2021; Song et al., 2023), popularity (e.g., Haim et al., 2018), stereotypes (e.g., Lombardi Vallauri, 2021), and topic (Schaewitz et al., 2020)—are purposefully manipulated and used by online propagandists to alter the attitudes and behaviors of their targeted audience.

Investigations focusing on the second factor explore how the very design of SNPs affects the way information is accessed, processed, and finally evaluated. Indeed, such environments are characterized by an overabundance of informational stimuli constantly competing for users' attention—a condition that impairs the ability to process and evaluate information analytically (Pittman and Haley, 2023) and that, as such, can be exploited by online propagandists to enhance the circulation of their messages (e.g., Islam et al., 2020; Apuke and Omar, 2021; Sanderson et al., 2022).

Finally, when exploring the role recipients' characteristics have in affecting online propaganda persuasive outcome, demand-side scholars tend to concentrate on those aspects that are in direct relation to the piece of information evaluated, namely receivers' “prior attitudes” and “evaluative beliefs” toward the issue addressed by propaganda messages (e.g., Ma et al., 2019; Hameleers et al., 2020; Rhodes, 2022), as well as the “motivation” (e.g., Van Bavel and Pereira, 2018; Stanley et al., 2020) and “effort” (e.g., Pennycook and Rand, 2019) these actors put into processing the information they are exposed to.

To provide compelling explanations on how these factors affect the evaluation process of online propaganda material and determine a voluntary change of attitudes and/or behavior in the targeted audience, researchers often resort to the concept of *heuristic reasoning*. This draws from the Dual Process Models of Cognition—a theory that envisages the existence of two distinct but interdependent systems that regulate the thinking process (Gilovich et al., 2002; Vaisey, 2009; Kahneman, 2011; Lizardo et al., 2016; Bryanov and Vziatysheva, 2021). Despite the ongoing debate on the specific characteristics and relations between these two systems (Cerulo et al., 2021), scholars agree on their basic functioning: one system is intuitive and does not require controlled attention during information processing, while the other is deliberate and necessitates more cognitive resources to perform mental tasks (Gilovich et al., 2002).

Because of its speed and (low) cognitive-energy demands, the former system is often employed in situations where reasoning capacity is impaired—such as in the case of high informational load (Ayres and van Gog, 2009)—or in cases of high uncertainty—when fast solutions are preferred (Kahneman et al., 1982). Though satisfactory for reaching immediate goals, this system is subject to systematic bias, as it relies on the uncritical application of preexisting knowledge structures (i.e., heuristics) rather than an in-depth analysis of the information received (DiMaggio, 1997). Therefore, the reliance on this system when processing information can lead to flawed evaluations and behavior (Gilovich et al., 2002).

Given its premises, the Dual Process Model—and, in particular, the concept of heuristic processing—has been largely employed by demand-side scholars to theoretically postulate and then empirically explore the cognitive mechanisms underpinning the assessment of online propaganda. Regardless of the specific factor (treatments, settings, or receivers) or outcome (evaluations or sharing behavior) investigated, empirical discoveries offered by this strand of the literature indicate that heuristic reasoning plays an important role in the persuasion process prompted by online propaganda (Bryanov and Vziatysheva, 2021).

Though compelling, these results are affected by some important limitations that mostly concern the way these cognitive mechanisms are conceived and, consequently, assessed. Overall, studies building on the Dual Process Model framework tend to have a universal approach to cognition, meaning that they conceive cognitive processes as common procedures shared by all individuals regardless of their socioeconomic, cultural, ethnic, or political background (Lamont et al., 2017; Kuo and Marwick, 2021). However, such factors have been demonstrated to play an important role in shaping these processes, as they make cognitive referents more accessible or prominent than others during information assessment and decision-making (Bruch and Feinberg, 2017; Lamont et al., 2017).

Neglecting these elements could limit result interpretation and, thus, potentially hinder the overall understanding of the mechanisms underlying complex social phenomena—especially if they involve communication practices, as in the case of online propaganda. Indeed, numerous studies have highlighted how communication is affected by sociocultural factors, that do not only influence the way a message is processed and evaluated, but also how that very message is designed and conveyed (e.g., Servaes, 1989; Lareau, 2011; Samovar et al., 2016).

Notwithstanding such arguments, demand-side investigations of online propaganda impact tend to neglect sociocultural differences in both treatment design (Kuo and Marwick, 2021) and effect assessment (Guess and Lyons, 2020). Engaging with sociocultural factors would, therefore, help this strand of the literature to relax the homogeneity assumptions that currently characterize its investigations—as these are rarely justifiable when compared to real-world scenarios characterized by high intrinsic diversity, both in terms of propaganda material and recipients' characteristics.

## 6. Discussion: bridging online propaganda literature

Employing the GP framework as a common frame of reference to analyze the disconnected body of literature on online propaganda offers a dual advantage. Firstly, it facilitates the identification of variations among different strands of the literature in terms of the specific dimensions and components addressed, thus supporting the development of a structured overview of the state-of-the-art. Secondly, it enables the placement of gaps and limitations of each strand within these dimensions and components.

In this sense, the adoption of a common framework proves beneficial not only in clearly identifying the shortcomings of both supply- and demand-side strands, but also in exploring avenues to bridge such strands. Indeed, this systematization has highlighted how this bridging process can be streamlined: on the one hand, by comprehending how the identified differences can be translated into valuable insights that each strand can employ to overcome its limitations; on the other hand, by exploring how the contributions offered by other research programs that focus on the interaction between cognitive and sociocultural factors can provide additional support to address such shortcomings. This integration of diverse perspectives would reduce fragmentation by harmonizing currently disjointed theoretical conceptualizations and empirical findings. Moreover, it would advance the research agenda on online propaganda by explicitly considering the interplay between its cognitive and social components.

### 6.1. Getting the dialogue going: how each strand of literature on online propaganda can contribute to the development of the other

Engaging in a constructive dialogue between the supply-side and demand-side strands of online propaganda literature can be advantageous for both, as each strand has the potential to offer valuable research tools to the other, aiding in the resolution of certain limitations that impact their respective areas.

As previously discussed, the supply-side strand is preoccupied with the socio-political factors leading to the emergence and endurance of online propaganda. To this aim, it focuses its investigations on the political actors involved in the production and dissemination of such political content and the strategies they employ to amplify its circulation. Notwithstanding its compelling findings, this strand remains primarily descriptive, with a disproportioned focus on the “speakers” component and some untested assumptions on the persuasive outcomes this type of communication generates (Jungherr et al., 2020; Camargo and Simon, 2022).

To better understand the persuasive power of online propaganda and its resulting effects, it is essential for this strand to consider the “receivers” component and the cognitive mechanisms that underpin their information processing and evaluation. This does not imply replicating or fully incorporating the research conducted by the demand-side strand, but rather acknowledging its existence and drawing from it when discussing online propaganda outcomes. By doing so, these contributions can be better positioned within the existing literature and in relation to the demand-side strand. Additionally, this would also enable a reevaluation of the (potential) social harm associated with online propaganda, as some have raised concerns about the validity of claims based solely on computationally intensive methods applied to social media data, without reference to real-world populations (Rauchfleisch and Kaiser, 2020; Camargo and Simon, 2022).

Conversely, the demand-side strand delves into the persuasion processes triggered by online propaganda, assessing its impact on users' evaluations and behavior. As such, it investigates how individuals process and respond to these persuasive stimuli to identify the cognitive mechanisms underlying such reactions.

Though rich in insightful findings on the outcomes produced by this kind of communication, this account as well is affected by some important limitations. By primarily focusing on the “recipients” component, investigations belonging to the demand-side strand tend to systematically overlook the socio-political context in which propaganda is created and disseminated when assessing its impact on the targeted audience. Furthermore, such assessments often adopt a universalistic perspective on cognition, assuming that cognitive processes underlying the evaluation of political content are universally applicable—an assumption that completely neglects the impact sociocultural factors have on cognition (Lamont et al., 2017) and, thus, on the evaluation of online propaganda content (Rampersad and Althiyabi, 2020).

However, drawing from the insights offered by the supply-side strand could help to partially mitigate such shortcomings and achieve a better interpretation of experimental results. The descriptive findings provided by the supply-side literature regarding, on the one hand, the actors involved in online propaganda production (including the political settings in which they operate) and, on the other hand, the consumption and diffusion patterns of propaganda on SNPs, can help contextualize the effects of exposure to online propaganda. Indeed, by drawing from these contributions, it would be possible to delve into the specificities of the socio-political context in which online propaganda is produced and disseminated, facilitating the development of more consistent stimuli tailored to specific subpopulations under investigation. Moreover, knowledge of the circulation and consumption patterns of online propaganda can be extremely useful for verifying the intuitions guiding the experimental assessment of the effect this communication practice has on users' judgments and behavior, as these real-world observations can be used as ground rules to compare (and substantiate) experimental findings with.

Overall, considering how each strand of the literature can build on the other to overcome its limitations is the first important step toward bridging online propaganda literature. However, this would still not be sufficient to address all the shortcomings affecting both strands, particularly those related to the neglect of sociocultural factors when investigating the functioning and outcomes of online propaganda. As previously mentioned, these factors profoundly affect human communication practices, including the way people process and evaluate messages (Martin and Nakayama, 1999). Therefore, they should be acknowledged when investigating online propaganda, as they can help interpret and contextualize findings, and provide a more comprehensive understanding of the underlying mechanisms that produce them.

### 6.2. How cultural and cognitive sociology can further bridge and enhance online propaganda scholarship

To address these remaining shortcomings, I argue that drawing from sociology—in particular, from its cultural and cognitive branches—would be beneficial, as this literature provides important insights and research tools to disentangle and evaluate the role played by supra-individual factors in the production, distribution, consumption, and evaluation of online propaganda. Indeed, utilizing the contributions provided by this scholarship would help in better contextualizing the settings in which online propaganda is produced and supplied, as well as advancing the understanding of the mechanisms underlying online propaganda outcomes.

By exploring and comparing the role that history, language, cultural system, and social differentiation play in the production and distribution of online propaganda across different contexts, it would be possible to evaluate how these factors influence and differentiate online propaganda dynamics. This contextualization could potentially aid the interpretation of existing findings that show significant variations in terms of speakers involved and persuasion strategies employed across settings (Bradshaw and Howard, 2018), since salience, credibility, and effectiveness of certain political actors or messages are likely to (also) depend on the sociocultural specificities characterizing the society in which this form of communication takes place (Rampersad and Althiyabi, 2020).

Furthermore, an improved understanding of the outcomes and underlying mechanisms of online propaganda can be achieved by examining how sociocultural factors interact with cognition and mutually shape each other, thereby influencing the evaluation of online propaganda messages. Contributions from cognitive and cultural sociology have, indeed, demonstrated that the cognitive mechanisms involved in information processing and decision-making are not universally applicable, as they are influenced by cultural repertoires—namely “the available schemas, frames, narratives, scripts and boundaries that actors draw on in social situations” (Lamont et al., 2017, p. 866). These repertoires are distributed unevenly among individuals who share the same national membership due to their transmission and diffusion by specific intermediaries—such as religious leaders, political parties, and media outlets—which may vary in prominence across different social groups (Rosen, 2017; Rucks-Ahidiana, 2022). Social differentiation thus plays a crucial role in determining the accessibility of cultural repertoires to different groups, as differently structured social environments enable certain cultural references to be more readily available to some individuals than others (Lamont et al., 2017). Consequently, this has an impact on how messages are processed, interpreted, and evaluated—a crucial insight that should be taken into account by scholars who are interested in identifying the different components underpinning the evaluation of online propaganda.

Therefore, engaging with sociocultural factors when investigating the functioning and outcomes of online propaganda would have the dual advantage of helping overcome the limitations affecting both research strands and further bridging the overall literature on online propaganda.

By considering sociocultural factors, researchers can move beyond a descriptive—and often Anglocentric—understanding of online propaganda production and distribution, reaching more comprehensive conclusions regarding the prominence and significance of specific platforms, actors, and diffusion patterns across different national and subnational context (Camargo and Simon, 2022). Supply-side scholars can, therefore, enhance their findings by incorporating these factors into their investigations, thus also responding to recent calls numerous researchers have made to ground online propaganda studies in history, society, culture, and politics to avoid neglecting the role race, ethnicity, language, colonial legacy, gender, and class have in this phenomenon (e.g., Siegel, 2020; Kreiss, 2021; Kuo and Marwick, 2021; Nguỹen et al., 2022).

To do so, new theoretical and empirical analyses should focus on how social differentiation influences the dynamics of persuasive communication. For instance, when examining consumption and diffusion patterns of online propaganda on SNPs, researchers could explore whether the most prominent accounts in these networks exhibit specific characteristics that symbolize their belonging to a trusted and authoritative group within the socio-cultural context. Similarly, they could expand upon insights from political bot research by investigating whether the display of cultural-specific traits and codes, such as jargon, religious symbols, or impersonation of community members, aids automated accounts in spreading propaganda content on social networking platforms and building trust among their targeted audience.

Acknowledging the influence of sociocultural factors would also enable researchers to move beyond a universal and static perspective of the cognitive mechanisms involved in the evaluation of online propaganda. By recognizing that cultural repertoires shape cognition and that their distribution varies among diverse social groups, demand-side scholars can achieve a more comprehensive understanding of how sociocultural factors shape cognitive processes and subsequently impact attitudinal and behavioral outcomes produced by this form of communication. Indeed, including sociocultural factors in the formulation and evaluation of the mechanisms underpinning online propaganda effects would allow researchers to relax the homogeneity assumption previously discussed and provide stronger causal explanations for the variations observed among recipients in terms of their response to online propaganda. To achieve such an objective, researcher could, for example, collect sociocultural information along with the “more classical” demographics during experimental assessments of online propaganda's impact on users' evaluations and behavior. This would allow for the estimation of heterogeneous treatment effects to assess whether individuals with similar sociocultural backgrounds exhibit consistent responses to specific propaganda stimuli.

By employing this approach, it would be possible to gain a deeper understanding of whether susceptibility to online propaganda persuasion strategies is influenced by supra-individual characteristics. This would provide scholars with new lens through which to explore and analyze patterns of vulnerability, thus further developing our understanding of online propaganda impact on individuals.

The acknowledgment and inclusion of sociocultural factors when investigating online propaganda functioning would further bridge the supply- and demand-side strands of literature. Indeed, adopting a consistent research approach that incorporates sociocultural factors in both conceptualizations and empirical assessments would facilitate the dialogue between the two strands, fostering meaningful knowledge exchange about this type of communication.

Moreover, adopting a sociocultural approach would serve as a cautionary measure against generalized—and, sometimes, oversimplified—policy proposals aimed at countering online propaganda. Whether these proposals target the persuasive influence of propaganda (e.g., debiasing treatments) or seek to regulate its dissemination (e.g., SNPs and content regulations), I argue that considering the sociocultural specificity of different target audiences is crucial.

For instance, scholars developing debiasing treatments that target cognition (e.g., Dai et al., 2021; Lewandowsky and van der Linden, 2021) would benefit from the inclusion of sociocultural factors in their analyses as this would allow them to develop more effective debiasing stimuli that account for the diversity characterizing online propaganda audience. Indeed, by taking into account that individuals' sociocultural background affects the availability of cultural repertoires and, thus, the cognitive mechanisms underlying information-processing and decision-making, researchers would be able to design tailored debiasing treatments that work best for specific socio-cultural groups (Sya'bandari et al., 2022).

Similarly, regulations that prescribe content and behavior limitations on SNPs^12^—and envisage social media companies' responsibilities in implementing such restrains—should be designed by considering how sociocultural factors interact with the production and consumption of online propaganda. On the one hand, this means acknowledging the specific use that political actors and citizens make of these platforms in different contexts, by identifying and assessing, for example, the most prominent platforms for political communication, the extent to which automation is employed, the type of actors involved in dubious communication activities, and the characteristics of diffusion and consumption patterns. On the other hand, it means considering the broader characteristics of the online media ecosystem, including how its ownership structure and the power dynamics that emerge from its interaction with the offline social world influence the production and consumption of such political content (Fuchs, 2019).

Overall, integrating the demand- and supply-side strands of research on online propaganda would enhance our knowledge of how propaganda impacts individuals' cognitive processes and behavior within specific socio-political contexts. By incorporating insights from both perspectives, researchers can conduct more rigorous and comprehensive analyses, making valuable contributions to the field. Moreover, by drawing on the insights offered by cultural and cognitive sociology, an improved understanding of the outcomes and underlying mechanisms of online propaganda can be achieved. Embracing a cultural perspective in the study of online propaganda would contribute to a more holistic understanding of this communication phenomenon. Indeed, it would allow scholars to explore factors that account for significant variations among diverse social groups, which often stem from existing patterns of inequality that result in uneven distributions of material and cultural resources.

## Author contributions

The author confirms sole responsibility for the study conception and design, analysis and interpretation of bibliographic sources, and manuscript preparation.

## Footnotes

^1^Here is an overview of all the components in relation to their respective dimensions: actors (speakers and receivers), treatments (topics, content and media), outcomes (attitudes, behaviors, emotions and identities) and settings (competition, space, time, process, and culture) (Druckman, 2022).

^2^As outlined in the following section, some authors belonging to the supply-side strand of the literature have discussed online propaganda “receivers” and “outcomes” (e.g., Woolley and Howard, 2018). However, they have done so in a descriptive way, by limiting their considerations to the accounts engaging the most with this kind of political content or by speculating on its effects without formulating empirically testable propositions. For these reasons, these components/dimensions are not considered to be the core focus of this approach.

^3^It is important to note that studies exploring how the online media system and its ownership structure affect the production, circulation, and consumption of (political) information on SNPs (e.g., Fuchs, 2018; Marmura, 2020; Arayankalam and Krishnan, 2021) are not considered to be part of this specific body of literature. Inasmuch as they explore the reproduction of power structures in online environments and their impact on news media and communication at large, these contributions discuss online propaganda as a byproduct of such dynamics (like echo chambers, polarized conversations, etc.) rather than directly addressing its function and role as a political practice. For this reason, they are not addressed in this paper as they exceed the scope of this analysis.

^4^As in the famous case of the Macedonian teenagers who created and disseminated pro-Trump material for profit in 2016 (Subramanian, 2017).

^5^Defined as “user account[s] that ha[ve] been equipped with the features or software to automate interaction with other user accounts about politics” (Howard et al., 2018, p. 85).

^6^Building on the Agenda-Setting Theory (McCombs and Shaw, 1972; McCombs et al., 1997), these authors claim that the systematic use of computational tools allows political actors to bypass traditional gatekeepers of information and directly alter the salience of specific topics in the online public discourse. In this way, they drive media coverage toward political issues and narratives considered more advantageous for their faction, thereby shaping the political debate and influencing the electorate (Woolley and Howard, 2018).

^7^Woolley and Howard (2018, p. 244) indeed maintain that “making a causal claim from social media use to citizen engagement, trust in institutions, or voter sophistication is proving difficult to do even in countries for which there are significant amounts of data”.

^8^Some contributions belonging to the supply-side strand have provided interesting insights into the type of users who are more likely to be exposed to and engage with online propaganda on SNPs, by exploring the characteristics and the behavior of their social media accounts (Shao et al., 2017; Guess et al., 2019). However, such information is mostly descriptive, as it is based on online available data that, as such, does not explore the decision-making process underlying the displayed behavior.

^9^Intended as the extent to which there are reasonable grounds for believing information conveyed (Sundar, 2008).

^10^Understood as the quality of being trustable because of previous experience indicating so (Gauld and Williams, 2009).

^11^Intended as the ability of information-providers to convey correct information (Tate, 2009).

^12^For an overview of governance responses in the European system see Saurwein and Spencer-Smith (2020).
